# A 52-Year-Old Female with Multiple Swellings in Both Hands: Idiopathic Calcinosis Cutis

**DOI:** 10.7759/cureus.7471

**Published:** 2020-03-30

**Authors:** Durga Shankar Meena, Deepak Kumar, Gopal K Bohra, Mahendra Kumar Garg

**Affiliations:** 1 Medicine, All India Institute of Medical Sciences, Jodhpur, IND

**Keywords:** calcinosis cutis, idiopathic, diltiazem

## Abstract

Calcinosis cutis is a rare disorder characterized by deposition of insoluble calcium salts in skin and subcutaneous tissue. Depending upon the aetiology, there are five subtypes of calcinosis cutis described in the literature; dystrophic, metastatic, idiopathic, iatrogenic, and calciphylaxis. Idiopathic calcinosis cutis is rarely described in the literature. We herein report a 52-year-old female presented with calcinosis cutis in both hands. After ruling out metabolic and systemic causes of abnormal calcium deposition, the patient was diagnosed with idiopathic calcinosis cutis. The patient was prescribed oral diltiazem (1 mg/kg). Despite limited treatment success in idiopathic calcinosis cutis, it is imperative to differentiate it from other disorders of calcium metabolism which can be managed by treating the underlying condition.

## Introduction

In calcinosis cutis, there is an abnormal deposition of calcium phosphate in the skin. The pathophysiology of this abnormal deposition was first described by Virchow in 1855. Dystrophic, metastatic, iatrogenic, idiopathic and calciphylaxis are the five subtypes of calcinosis cutis depending upon the underlying aetiology [1]. Idiopathic calcinosis cutis is an uncommon variant, associated with normal calcium metabolism and without tissue injury. Idiopathic calcification of scrotum, subepidermal calcified nodule and tumoral calcinosis are the various forms of idiopathic calcinosis cutis described in the literature [2-4]. Our report highlights a rare case of idiopathic calcinosis cutis in a 52-year-old female presented with chalky white swelling of several fingertips.

## Case presentation

**Figure 1:**
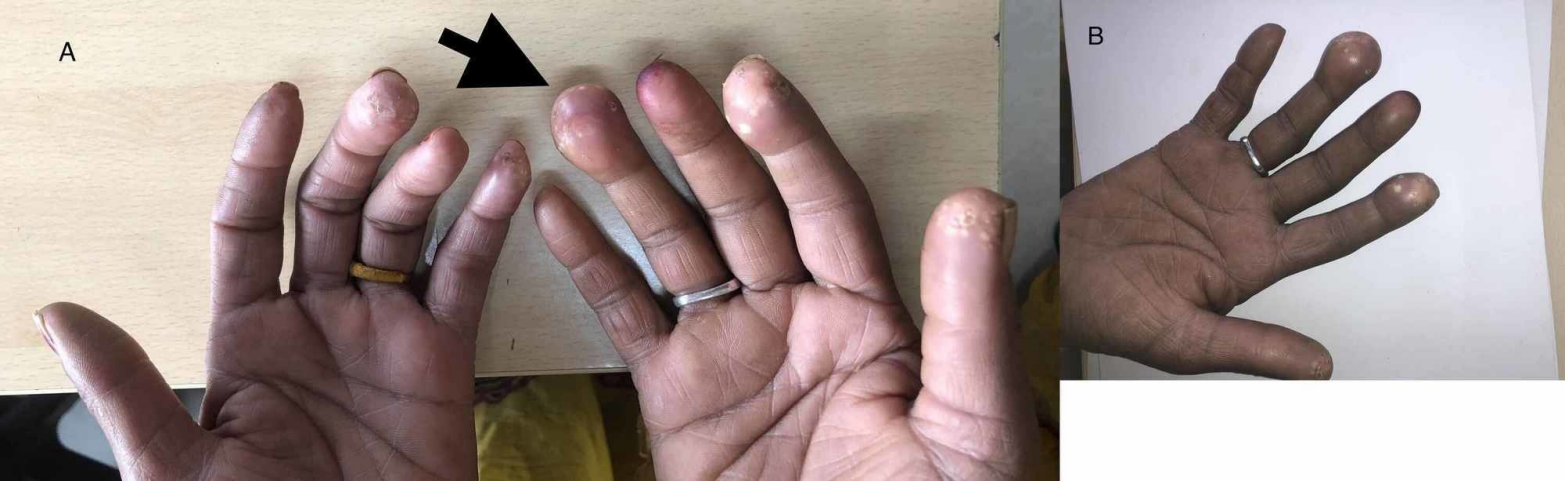
(A, B) Showing grouped white to yellowish papules on the finger pads (black arrow)

**Figure 2:**
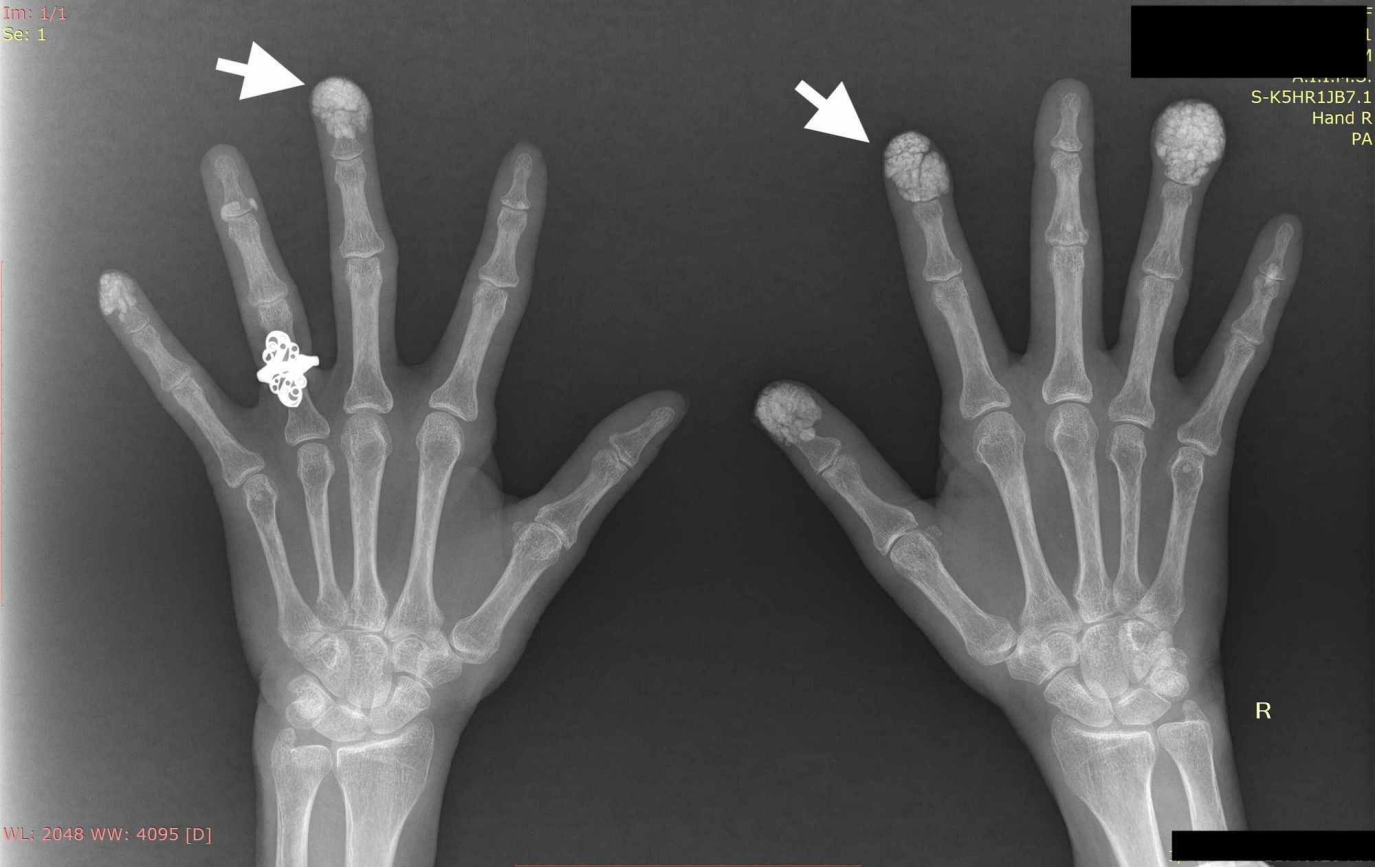
Radiograph of hands showing multiple calcifications within subcutaneous tissue in finger pads (white arrows)

A 52-year-old female with no significant past medical history presented with complaints of multiple yellowish-white swelling over fingertips for the last two years (swellings were bilateral involving multiple fingers). She had no other systemic complaints. Her family history and social history was unremarkable. Clinical examination revealed multiple, firm to hard, white to yellow papulonodular swellings on the finger pad of thumb, index and ring finger in right hand, little and middle finger of left hand (Figure 1A, 1B). With this clinical picture differential diagnosis of gouty tophi and calcinosis cutis were kept. On further evaluation, her radiograph of bilateral hand revealed multiple, heterogeneous soft tissue calcification in finger pads suggestive of calcinosis cutis (Figure 2). She was investigated further to find the cause and her liver function, renal function tests, serum electrolytes, C-reactive protein, and erythrocyte sedimentation rate were within normal limit. Serum calcium (9.8 mg/dl), magnesium, phosphate, uric acid, vitamin D3, thyroid function and parathyroid hormone (58 ng/ml, normal 10-75 ng/ml) levels were within the normal range. In addition, her 24-hr urinary calcium was also within the normal range. The workup for connective tissue disorders was negative (Antinuclear antibodies, anti-Scl-70, anti-Ro, anti-La, anti-Jo-1 antibodies were negative). Our patient refused skin biopsy. After ruling out metabolic causes, autoimmune disorders and malignancy, we made the final diagnosis of idiopathic calcinosis cutis. The patient was prescribed oral diltiazem (60 mg, 1 mg/kg) and was followed for the next three months. Though her lesions were not progressive, there was no significant improvement after diltiazem therapy.

## Discussion

Calcinosis cutis is characterized by deposition of insoluble calcium salts in the subcutaneous and cutaneous tissue. Dystrophic calcification is the most common type of calcinosis cutis and associated with underlying tissue damage. Dystrophic calcification is seen in various connective tissue disorders (scleroderma, lupus erythematosus, mixed connective tissue disorder, dermatomyositis). Dystrophic calcification can be seen rarely with Werner syndrome, Ehlers-Danlos syndrome, panniculitis, basal cell carcinoma and cysticercosis. Metastatic calcification occurs in patients with abnormal calcium/phosphate metabolism (chronic kidney disease, hyperparathyroidism, milk-alkali syndrome, sarcoidosis and malignant neoplasm). Calciphylaxis, iatrogenic and idiopathic are other presentation of calcinosis cutis. Calciphylaxis is a form of calcific vasculopathy usually associated with patients of end-stage renal disease, which involves small and medium-size vessels in dermis.

Idiopathic calcinosis cutis is rarely reported in the literature [5-7]. It occurs without any tissue damage or abnormal calcium or phosphate metabolism. There are three subtypes of idiopathic calcinosis described in the literature; scrotal calcinosis, familial tumoral calcinosis and subepidermal calcified nodules. The underlying pathophysiology behind the abnormal deposition of calcium salts in skin is unclear. It has been hypothesized that abnormal metabolism of gamma carboxy glutamic acid (GIa) is responsible for abnormal calcium deposition in subcutaneous tissues, with increased production of GIa is attributed to soft tissue calcification [8]. A mutation in the gamma-glutamyl carboxylase gene is also reported to cause aberrant calcification in dermal fibroblast [9].

Treatment of calcinosis cutis is challenging. The limited role of warfarin, diltiazem, bisphosphonates, probenecid, colchicine, aluminium and magnesium antacids has been described in the literature. Previous reports have conflicting results of diltiazem in idiopathic calcinosis cutis with some reports showing significant resolution with long-term therapy [10,11]. It has been postulated that being a calcium channel blocker, diltiazem inhibits the calcium accumulation into the cells. The dose of diltiazem was varied from 1 mg/kg to 3 mg/kg (up to 330 mg) in previous cases. We started from 60 mg and increased the dose up to 200 mg without any significant result. The indications for surgical removal are ulceration, infection, pain and functional impairment. Our patient had none of these complications, so we decided to observe the patient with close follow-up.

## Conclusions

In conclusion, our report illustrates the rare presentation of calcinosis cutis. Extensive evaluation to rule out any potential abnormalities of calcium and phosphate metabolism, connective tissue disorders, renal dysfunction, and malignancy should be done. Though the role of medical management is limited in idiopathic calcinosis cutis, the possibility of correctable/secondary causes should be sought in patients presented with abnormal soft tissue calcification. There are recent reports which showed promising results with diltiazem therapy however our patient did not show any improvement. Further studies will be required to establish the role of calcium channel blockers in the treatment of calcinosis cutis.

## References

[1] Reiter N, El-Shabrawi L, Leinweber B, Berghold A, Aberer E (2011). Calcinosis cutis. Part I: diagnostic pathway. J Am Acad Dermatol.

[2] Nico MM, Bergonse FN (2001). Subepidermal calcified nodule: report of two cases and review of the literature. Pediatr Dermatol.

[3] Leung YY, Lai R (2011). Tumoral calcinosis: a case report. J Orthop Surg.

[4] Holliday AC, Clos A, Kelly B (2014). Firm papules on the penis and scrotum. Dermatol Online J.

[5] Jatana SK, Negi V, Das S (2012). A case of idiopathic calcinosis cutis. Med J Armed Forces India.

[6] Ferdaus-Kamudin NA, Mohamed-Haflah NH (2018). Multiple scattered and small lesions of lower limbs: idiopathic calcinosis cutis: a case report. Malays Orthop J.

[7] Alsaif F, Abduljabbar AM (2017). Unilateral idiopathic calcinosis cutis: a case report. Case Rep Dermatol.

[8] Van Summeren MJ, Spliet WG, Van Royen-Kerkhof A, Vermeer C, Lilien M, Kuis W, Schurgers LJ (2008). Calcinosis in juvenile dermatomyositis: a possible role for the vitamin K-dependent protein matrix Gla protein. Rheumatology.

[9] Okubo Y, Masuyama R, Iwanaga A (2017). Calcification in dermal fibroblasts from a patient with GGCX syndrome accompanied by upregulation of osteogenic molecules. PLoS One.

[10] Singh J, Paliwal VK, Bhargava P, Mathur DK (2017). Idiopathic calcinosis cutis universalis treated successfully with oral diltiazem-A case report. Pediatr Dermatol.

[11] Abdallah-Lotf M, Grasland A, Vinceneux P, Sigal-Grinberg M (2005). Regression of cutis calcinosis with diltiazem in adult dermatomyositis. Eur J Dermatol.

